# 

**Published:** 1905-12

**Authors:** 


### CONTENTS.

ORIGINAL COMMUNICATIONS—

The Treatment of Corneal Ulcers by the General Practitioners. By Clarence Porter Jones, M.D., Newport News, Va. .......... 577
Refilling Prescriptions. By R. B. Kelley, Atlanta, Ga. .......... 581
Cancer—A Few Suggestions. By R. R. Kime, M.D., Atlanta, Ga. .......... 583
How I was Relieved of Locomotor Ataxia. By Clem C. Greene, M.D., Atlanta, Ga. .......... 585

EDITORIAL NOTES AND COMMENTS—

Peruna and the "Bracers" .......... 589

NEWS AND NOTES .......... 591

BOOK REVIEWS—

A Treatise on Diagnostic Methods of Examination. (Sahli) .......... 599
Hygiene and Public Health. (Whitelegge) .......... 600
A Non-Surgical Treatise on Diseases of the Prostate Gland and Adnexa. (Overall) .......... 600
Simon's Manual of Chemistry. (Simon) .......... 601
Diet in Health and Disease. (Friedenwald) .......... 602
A Manual of Chemistry, Inorganic and Organic. (Luff and Page) .......... 602
Physician's Pocket Account Book. (Taylor) .......... 603

CONTENTS CONTINUED .......... X

